# Number of Patient Encounters in Emergency Medicine Residency Does Not Correlate with In-Training Exam Domain Scores

**DOI:** 10.5811/westjem.2022.11.57997

**Published:** 2022-12-21

**Authors:** Michael W. Kern, Corlin M. Jewell, Dann J. Hekman, Benjamin H. Schnapp

**Affiliations:** * Mayo Clinic Health System – Northwest Wisconsin Region, Department of Emergency Medicine, Eau Claire, Wisconsin; † BerbeeWalsh Department of Emergency Medicine, University of Wisconsin School of Medicine and Public Health, Madison, Wisconsin

## Abstract

**Introduction:**

Emergency medicine (EM) residents take the American Board of Emergency Medicine (ABEM) In-Training Examination (ITE) every year. This examination is based on the ABEM Model of Clinical Practice (Model). The purpose of this study was to determine whether a relationship exists between the number of patient encounters a resident sees within a specific clinical domain and their ITE performance on questions that are related to that domain.

**Methods:**

Chief complaint data for each patient encounter was taken from the electronic health record for EM residents graduating in three consecutive years between 2016–2021. We excluded patient encounters without an assigned resident or a listed chief complaint. Chief complaints were then categorized into one of 20 domains based on the 2016 Model. We calculated correlations between the total number of encounters seen by a resident for all clinical years and their ITE performance for the corresponding clinical domain from their third year of training.

**Results:**

Available for analysis were a total of 232,625 patient encounters and 69 eligible residents who treated the patients. We found no statistically significant correlations following Bonferroni correction for multiple analyses.

**Conclusion:**

There was no correlation between the number of patient encounters a resident has within a clinical domain and their ITE performance on questions corresponding to that domain. This suggests the need for separate but parallel educational missions to achieve success in both the clinical environment and standardized testing.

## INTRODUCTION

Every year, emergency medicine (EM) residents take the in-training examination (ITE) administered by the American Board of Emergency Medicine (ABEM). This test is important, in part, due to its ability to predict who will pass the Qualifying Examination (QE).^1^,^2^ The QE is a critical part of ABEM’s certification process and, therefore, independent clinical practice.^3^ The ITE is designed to follow the EM Model of Clinical Practice (Model), which is based on an “extensive practice analysis of the specialty.”^4^ It has previously been shown in 2011 that no correlation exists between the total number of patient encounters during EM residency and ITE score.^3^ However, it is unclear whether any relationship exists between the number of patient encounters a resident has within a specific clinical domain during training and their ITE performance on questions that correspond to that domain. Should no relationship exist, it could call into question the utility the ITE might have in measuring whether a resident is progressing appropriately with regard to their clinical skills.

Kolb’s experiential learning theory would suggest that residents who have greater clinical exposure in a particular area (eg, cardiovascular complaints) should be able to better conceptualize and achieve a greater understanding of clinical concepts than simply reading about them alone, provided that they engage in patient follow-up, self-reflection, and/or facilitated feedback with attending physicians. If experiential learning theory were to apply to health professions education, residents with increased experience should theoretically perform better on ITE questions corresponding to that domain, as this test is meant to be a surrogate for the knowledge required to competently practice EM.^5^ Our purpose in this study was to determine whether there was a relationship between ITE performance within individual content domains of the Model and the number of patients seen during residency with chief complaints in each domain.

## METHODS

This project was deemed exempt quality improvement by the University of Wisconsin Health Sciences Institutional Review Board.

### Study Setting

We conducted the study at a three-year EM residency program situated within an urban, academic emergency department (ED) in the Midwest. The ED has 54 beds with a volume of approximately 60,000 patient visits annually. During the period of the study, the residency had 12 postgraduate year-1 positions available each year.

### Data Acquisition

In this study we used deidentified, first chief complaint data rather than downstream categorization (eg, final diagnosis, admitting diagnosis). We used chief complaints to identify the nature of the patient encounter as this data was available at the time of patient presentation, likely dictated most of the ED evaluation, and would not have been affected by changes in treatment identified during later stages of a patient’s hospital course. Residents were eligible for inclusion if they had graduated in three consecutive years between 2016–2021. All patient encounters from all years of training involving eligible EM residents were queried. To maintain anonymity, each resident was assigned a study identification number; the ID key was accessible only to the senior author, a member of the residency leadership team.

We excluded from analysis encounters where no chief complaint was listed or no resident was assigned. In cases where multiple residents were assigned to a single encounter, we designated the initial resident assigned to the encounter as the resident of record. This was done as the first resident is typically the most cognitively involved in determining the patient’s diagnostic and treatment strategy. The chief complaint for each encounter was determined by the patient’s primary nurse who cared for the patient in the ED initially, which is nearly always selected from a list of frequent chief complaints. Resident ITE scores across domains during the third year of training were taken from internal residency records.

### Data Analysis

A previously published list of common EM chief complaints had been compiled and independently categorized into one of 20 content domains correlating with the 2016 ABEM Model of Clinical Practice by two board-certified EM attending physicians.^4^ For all chief complaints appearing in our data that were not previously categorized, we repeated the same categorization process with two board-certified EM attending physicians at our institution. In both cases, if there was disagreement between the two reviewers, a third board-certified emergency physician was brought in to adjudicate. We categorized complaints in which a symptom was used as the descriptor and could potentially correspond to multiple organ systems (eg, chest pain) into domains based on what was most likely given the general experiences of the coding physicians, rather than into the “Signs, Symptoms, and Presentations” domain.

The ITE scores are reported by ABEM by domain according to the Model. We calculated Pearson’s correlation coefficient, Pearson’s coefficient of determination, and Spearman’s rank correlation along with 95% confidence intervals for each domain, comparing individual caseloads within each content area to the same individual’s ITE subscore percentages within that domain using SPSS (IBM Corporation, Armonk, NY). The Bonferroni correction for multiple comparisons was used to determine significance.

## RESULTS

We included in the analysis a total of 232,625 patient encounters from 69 residents in the analysis. Resident performance on the ITE is shown in Table 1. Correlation coefficients (Pearson’s) ranged from −0.12 to 0.28 for the different domains. Correlation coefficients for each topic’s clinical exposures and ITE scores, as well as their significance levels, are listed in Table 2. No significant correlations were identified after Bonferroni correction.

## DISCUSSION

The number of patient encounters within a certain domain showed no correlation to resident performance on the corresponding ITE domains. This is in line with previous studies that have demonstrated little relation between total number of patient encounters during residency and performance on formal testing.^3^ It has been demonstrated that differences exist between resident clinical exposure and the weight each domain is given on the ITE,^6^ but our study further suggests that a disconnect exists between the breadth of clinical encounters and ITE performance. This would suggest that program leadership should limit the use of ITE scores as a global assessment tool for a resident’s clinical progress and instead focus on those scores’ ability to predict success on the QE.

It appears that success in clinical practice does not imply success on standardized testing. This would provide an argument to maintain parallel, separate educational missions focused on each mission, as success in the clinical environment and passing the QE are both critical components of an emergency physician’s career after residency graduation. Requiring two separate missions would require a residency program to devote time to both, which could tax a program’s faculty. Alternatively, this dual focus would require a program to potentially rely on commercial products to provide the specific knowledge to do well on the ITE. Access to online question banks (Qbank LLC, Stockholm, Sweden) has been demonstrated to be beneficial,^7^ but their use may tax a residency’s financial resources. While it is possible that the breadth (or lack) of clinical experience in certain areas would direct a resident’s self-study practices, our study results suggest that this strategy may be suboptimal, at least as far as ITE study is concerned. Instead, residents would be best served with a broad study plan regardless of the range of their clinical encounters, which is in line with previous studies demonstrating the differences between residents’ patient care experiences and the blueprint provided by the Model.^6^,^8^ There remains room for further study to more clearly elucidate the link, if any, between clinical training and ITE performance.

Overall, our results appear to be in opposition to Kolb’s experiential learning theory, which would have suggested a more robust link between clinical experience and testing performance. There may be multiple reasons for this discrepancy. First, experiential learning theory relies heavily on reflection to translate experience into knowledge.^5^ On one hand, residents have multiple opportunities to reflect on cases during their clinical work, including documentation of the clinical encounter and feedback provided from faculty and other staff, as well as patient case logs as mandated by the Accreditation Coiuncil for Graduate Medical Education.^9^ On the other hand, it is possible that the amount of reflection for each case is low, particularly during busy shifts where the demands of patient care may limit the amount of time for case review and feedback. Reflection on clinical experiences also requires the identification of experiences as learning opportunities, which is often reliant on faculty and peers, and may not be recognized by trainees.^10^ Finally, there may be minimal to no dedicated time built into residency for residents to reflect; therefore, they must balance this against a busy schedule of other clinical and non-professional activities.

Another potential reason for the disconnect between actual clinical experiences and a corresponding ITE question is differences in medical content. It is possible that the topics discussed in the questions revolve around atypical presentations that are not seen frequently, if at all, during the span of a three- or four-year EM residency. If residents are not seeing certain pathologies (eg, scombroid poisoning) during their clinical shifts, then it would be unlikely that their clinical exposures would assist them on ITE questions. This does not imply that programs are not providing a comprehensive clinical experience to their residents, but rather that certain unavoidable gaps occur due to differences in communities served, geographical region, etc. For example, residents practicing in Wisconsin are unlikely to see a scorpion bite in their day-to-day clinical responsibilities, but this is identified as a critical topic in the Model. Therefore, program leaders should seek to identify areas in which potential clinical gaps exist and seek to devote extra time to these domains during their didactic conferences.

It is possible that ceiling effects are responsible for the overall lack of correlations we found and that residents who see a substantially lower number of patients in a particular domain would have lower ITE scores on that section. This may not have been captured by our data if the included residents did not fall below this threshold. However, programs perceiving a large deficit in clinical cases corresponding to a particular domain could review their own performance data to determine whether a significant deficit on their residents’ ITE score reports exists within that domain.

## LIMITATIONS

This study has several limitations. First, assessing case content by chief complaint could inappropriately categorize some presentations. For example, a patient presenting with a “behavior problem” (categorized under Psychobehavioral Disorders) could have anticholinergic toxicity because of an overdose (better categorized as a Toxicologic Disorder). While we considered using discharge or primary diagnosis instead of chief complaint to categorize our clinical exposures, we ultimately felt that this was inconsistent with the way EM is practiced. Additionally, some of the chief complaints of the encounters may have been categorized into the wrong domain due to errors on the part of the research team.

We used the 2016 Model of Clinical Practice, which informed the creation many of the ITEs administered during the years included in the study. Our study did not account for other factors that may have impacted a resident’s performance on the ITE, such as differences in the type and usage of exam preparatory materials, although study resources made freely available by the program were the same throughout the study period (Rosh Review, Los Angeles, CA). Effort in the clinical setting also may not translate to success on the ITE, as the test offers no direct disincentives for poor performance, and any incentives for success are program specific.^11^ Finally, this data was collected at a single site and, therefore, may be difficult to generalize to institutions with different clinical environments and test preparation resources.

## CONCLUSION

We found no significant correlation between resident clinical exposure and performance on the ITE. This study supports the concept that standardized test performance is not linked to performance in other areas and suggests the need for the creation of separate, parallel educational missions to achieve success in both areas.

## Figures and Tables

**Table 1:** Resident performance on the Emergency Medicine In-Training Examination.

Domain	Min	Max	Median	Mean
Signs, symptoms, and presentations 1.0	52.63%	100.00%	84.21%	82.38%
Abdominal and gastrointestinal disorders 2.0	66.67%	100.00%	83.33%	85.10%
Cardiovascular disorders 3.0	59.09%	95.45%	81.82%	81.29%
Cutaneous disorders 4.0	0.00%	100.00%	50.00%	58.72%
Endocrine, metabolic, and nutritional disorders 5.0	40.00%	100.00%	95.45%	86.39%
Environmental disorders 6.0	33.33%	100.00%	83.33%	80.74%
Head, ear, eye, nose, and throat disorders 7.0	40.00%	100.00%	75.00%	78.21%
Hematologic disorders 8.0	0.00%	100.00%	85.71%	81.56%
Immune system disorders 9.0	33.33%	100.00%	75.00%	76.93%
Systemic infectious disorders 10.0	11.69%	100.00%	73.33%	74.42%
Musculoskeletal disorders (non-traumatic) 11.0	57.14%	100.00%	85.71%	86.97%
Nervous system disorders 12.0	50.00%	100.00%	81.82%	78.34%
Obstetrics and gynecology 13.0	37.50%	100.00%	83.33%	80.68%
Psychobehavioral disorders 14.0	37.50%	100.00%	85.71%	81.09%
Renal and urogenital disorders 15.0	42.86%	100.00%	84.52%	80.51%
Thoracic-respiratory disorders 16.0	58.82%	100.00%	77.78%	79.53%
Toxicologic disorders 17.0	45.45%	100.00%	81.82%	78.51%
Traumatic disorders 18.0	40.91%	95.45%	76.19%	74.09%
Procedures and skills 19.0	47.06%	100.00%	77.78%	77.18%
Other components 20.0	0.00%	100.00%	83.33%	79.13%

**Table 2:** Correlations between number of patient encounters and in-training exam scores for each of the American Board of Emergency Medicine Model of Clinical Practice domains.

Domain	Pearson’s Correlation	R^2^	95% confidence interval	P value	Spearman’s Correlation	95% confidence interval	P value	Case total
Abdominal and gastrointestinal disorders	0.14	0.02	−0.10 – 0.37	0.24	0.15	−0.10 –0.37	0.23	40,819
Cardiovascular disorders	−0.03	0.00	−0.26 – 0.21	0.84	−0.01	−0.25 –0.24	0.97	22,918
Cutaneous disorders	−0.05	0.00	−0.28 – 0.19	0.7	−0.09	−0.33 –0.16	0.46	3,444
Endocrine, metabolic, and nutritional disorders	0.06	0.00	−0.18 – 0.29	0.64	0.07	−0.17 –0.31	0.54	1,326
Environmental disorders	0.05	0.00	−0.19 – 0.28	0.7	0.08	−0.17 –0.31	0.54	1,198
Head, ear, eye, nose, and throat disorders	0.25	0.06	0.02 – 0.46	0.03	0.30	0.07 – 0.51	0.01	10,516
Hematologic disorders	−0.04	0.00	−0.27 – 0.20	0.74	0.04	−0.20 – 0.28	0.72	729
Immune system disorders	−0.02	0.00	−0.25 – 0.22	0.88	0.02	−0.22 – 0.26	0.86	1,860
Musculoskeletal disorders (non-traumatic)	−0.25	−0.06	−0.45 – −0.01	0.04	−0.23	−0.45 – 0.01	0.05	21,984
Nervous system disorders	−0.07	0.00	−0.30 – 0.17	0.55	−0.18	−0.40 – 0.06	0.13	22,299
Obstetrics and gynecology	−0.04	0.00	−0.27 – 0.20	0.77	0.03	−0.21 – 0.27	0.80	1,377
Other components	0.03	0.00	−0.21 – 0.26	0.81	0.07	−0.18 – 0.30	0.59	7,172
Procedures and skills	−0.20	0.04	−0.41 – 0.04	0.1	−0.25	−0.46 – −0.01	0.04	2,591
Psychobehavioral disorders	0.28	0.08	0.05 – 0.48	0.02	0.30	0.06 – 0.51	0.01	8,832
Renal and urogenital disorders	−0.04	0.00	−0.27 – 0.20	0.73	−0.05	−0.28 – 0.20	0.71	5,019
Signs, symptoms, and presentations	−0.03	0.00	−0.26 – 0.21	0.81	−0.02	−0.26 – 0.23	0.90	9,019
Systemic infectious disorders	0.17	0.03	−0.07 – 0.39	0.17	0.14	−0.11 – 0.37	0.26	11,566
Thoracic-respiratory disorders	0.10	0.00	−0.14 – 0.33	0.39	0.08	−0.17 – 0.32	0.51	19,608
Toxicologic disorders	−0.04	0.00	−0.28 – 0.19	0.72	−0.05	−0.28 – 0.20	0.71	3,338
Traumatic disorders	−0.12	−0.01	−0.35 – 0.11	0.31	−0.16	−0.38 – 0.09	0.20	37,010
